# Editorial: Regulation of and by the Plant Cell Wall

**DOI:** 10.3389/fpls.2020.00513

**Published:** 2020-04-29

**Authors:** Xiaolan Rao, Laura E. Bartley, Georgia Drakakaki, Charles T. Anderson

**Affiliations:** ^1^College of Life Sciences, Hubei University, Wuhan, China; ^2^Department of Biological Sciences, BioDiscovery Institute, University of North Texas, Denton, TX, United States; ^3^Microbiology and Plant Biology Department, University of Oklahoma, Norman, OK, United States; ^4^Research Institute for the Sustainable Humanosphere, Kyoto University, Kyoto, Japan; ^5^Department of Plant Sciences, University of California, Davis, Davis, CA, United States; ^6^Department of Biology, The Pennsylvania State University, University Park, PA, United States

**Keywords:** plant cell wall, transcriptional regulation, cell wall biosynthesis, cell wall modification, vesicle-mediated trafficking, polysaccharide transport

The cell wall encapsulates plant cells and fundamentally influences their properties. Wall components and interactions vary among the major plant clades, throughout plant development, among different cell types, and even in response to external and internal stimuli. In addition to their fundamental roles in plant development and physiology, plant cell walls represent the most abundant terrestrial carbon sink and, thus, a key alternative to fossil carbon utilization for energy and materials (Youngs and Somerville, 2012).

This Frontiers in Plant Science virtual issue on “Regulation of and by the Plant Cell Wall” consists of 14 publications, including 8 reviews and 6 original research articles, which fall into three general topics: cell wall composition, synthesis, and modification; transcript-level regulation of cell wall synthesis; and the cell biology of walls. Understanding the regulation of plant cell wall biosynthesis and modification is fundamental to understanding plant development and provides insights for biotechnological innovation for the bioenergy, bio-product, and forage industries. As summarized below, each paper ties into the theme of regulation, gathering evidence of either direct regulation of cell wall components, homeostasis of cell wall composition, compensation among cell wall properties, or feedback of cell wall properties to plant physiology and development. Several papers present methods related to probing cell wall properties or biology.

## Cell Wall Composition, Synthesis, and Modification

The most abundant plant cell wall polymer, cellulose, is a partially crystalline polymer and is thought to be the main load-bearing component of walls. The structure and arrangement of cellulose contributes to the mechanical properties of the cell wall and to anisotropic cell growth. Rongpipi et al. provide a detailed review of methods for physical characterization of cellulose microfibrils at different scales, including structural parameters such as shape, degree of polymerization, crystallinity, and spatial organization. The authors' descriptions of the practical consideration of techniques such as x-ray diffraction, x-ray scattering, spectroscopy, and microscopy will certainly be of use to researchers. In related research, Mazarei et al. use transgenic approaches to characterize two cellulose synthase genes of switchgrass, *PvCesA6*, a predicted primary wall synthase, and *PvCesA4*, a predicted secondary wall synthase. (Secondary cell walls are deposited inside primary walls in many cell types after growth cessation). Both down-regulation and overexpression lines lead to reduced cellulose content and crystallinity and reduced plant stature. Several lines have modified amounts of non-cellulosic cell wall polymers such as lignin and xylan, extending the evidence for functional compensation among cell wall polymers.

The polyphenolic, lignin, has been a subject of intensive research due to its role in preventing the efficient release and utilization of cell wall polysaccharides during the processing of plant biomass, and conversely as a valuable chemical precursor. Xie et al. review the transcriptional regulation, biosynthesis, and functions of lignin in growth and defense. While focusing mainly on well-studied pathways and networks from Arabidopsis, the authors also incorporate newer information about alternative pathways in other species. The review highlights the pleiotropic effects of lignin mutants, cataloging examples of growth defects that may connect lignin to different defense pathways.

Covalent modifications add further complexity to the composition and functions of cell wall polymers. This collection includes reviews of two polysaccharide-modifying enzyme groups, glycosyl O-acetyltransferases and polygalacturanases. Pauly and Ramírez provide a comprehensive update into the enzymes that O-acetylate the matrix polysaccharides xyloglucan, xylan, and pectin. The authors describe the protein families that conduct polysaccharide O-acetylation, compare similarities and differences in polysaccharide O-acetylation between plants and other organisms, and summarize evidence for roles in hormone signaling. Yang et al. highlight a major group of pectin-modifying enzymes, the polygalacturonases (PGs), which cleave pectic polysaccharides. Pectins are matrix polysaccharides in plant cell walls that are especially abundant in the eudicot primary walls. Yang et al. provide an overview of the functions of PGs in plant development, the classification and expression of PG family members, and their evolution across plant species, and describe potential regulatory functions of PGs in internal and external signaling.

## Transcript-Level Regulation of Cell Wall Synthesis

Hundreds of transcription factors have been implicated in regulating expression of genes related to cell wall biosynthesis and modification, either directly or indirectly. Two teams review recent advances in transcriptional and post-transcriptional regulation of enzymes involved in secondary cell wall synthesis in grass and woody plants. Rao and Dixon comprehensively discuss how the conservation and divergence of genes that regulate secondary wall deposition in grass and eudicot plants might be related to the distinct patterning and composition of the walls in these two plant groups. Similarly, Zhang et al. provide an update on understanding the regulation of secondary wall synthesis at the transcriptional level in woody species. They conclude that while many parallels exist, there appear to be added molecular complexities in the regulation of secondary wall deposition in trees relative to Arabidopsis.

This issue also reports new research on the transcriptional regulation of secondary wall synthesis in Arabidopsis, as well as crop and bioenergy species. In Arabidopsis, Olins et al. examine polymorphisms in the promoter of a secondary wall cellulose synthase, *AtCesA4*, that form the basis of an expression quantitative trait locus in a Bay-o X Shahdara recombinant inbred population. A single nucleotide polymorphism in a NAC transcription factor-binding motif reduces *AtCesA4* gene expression by 2-fold. Interestingly, cellulose content appears to be unaltered in lines with this variant, providing an example of cell wall homeostasis.

An important approach for dealing with the complexity of cell wall biology, three original research articles provide successful examples of exploring biological information derived from -omics data to identify secondary wall regulators. Furches et al. develop a multi-layered, network-based pipeline to search for novel secondary wall-related genes in poplar, combining datasets on gene co-expression, gene co-methylation, SNP correlations, and genome-wide association studies. Additional bioinformatics analysis supports a role in cell wall control for the transcription factor, PtGFR9, the Arabidopsis ortholog of which functions in drought-mediated growth inhibition. In another primarily bioinformatic study of woody species, Seyfferth et al. use an aspen transcriptome database to generate a co-expression network of genes involved in ethylene signaling, pursuing the role of ethylene in cambial growth. The aspen gene, *EIN3D*, is experimentally confirmed to function in ethylene signaling in Arabidopsis. Lastly, Zhao et al. develop a novel functional gene network in rice and mine it for cell wall regulators. They provide experimental evidence that a previously studied cell wall regulator, OsMYB61a, binds to the promoter of a grass-specific wall synthesis gene and that six out of 11 tested transcription factors function as novel regulators of secondary wall gene expression. The gene annotations generated by these studies provide additional wall regulatory candidates, and more generally, the analysis methods are likely to be useful for revealing crosstalk between biological pathways.

## Cell Wall Cell Biology

Post-translational cellular processes that mediate wall formation and rearrangement are another critical aspect of cell wall regulation. Cellulose synthases transit through the Golgi apparatus on the way to the plasma membrane, and many other glycosyltransferases reside in the Golgi. The highly dynamic nature of the endomembrane system makes it challenging to assign unequivocal roles to specific vesicle populations in the synthesis and assembly of the cell wall. Sinclair et al. summarize current research on the transport and deposition of cell wall components by the endomembrane system during cell division and growth. The authors describe the coordinated trafficking of cell wall polysaccharides and proteins, and wall biosynthetic and modifying enzymes, and discuss promising avenues to gain insights into the trafficking of structural polysaccharides to the apoplast. Related to cellular control of cell wall biophysics, Rui et al. spotlight the heterogeneous and dynamic three-dimensional arrangement of the cell walls of stomatal guard cells. Mutant studies coupled with microscopy reveal the role of cell wall dynamics in the opening and closing of guard cells for controlling gas diffusion at the plant surface. Badmi et al. report a new function for a putative calmodulin binding protein in poplar, PdIQD10, in cell wall biology, consistent with the multi-omic network analysis of Furches et al. Knocking down *PdIQD10* leads to larger poplar plants with increased cellulose content. Although the molecular mechanism by which PdIQD10 performs its cellular function remains elusive, the results suggest linkages between calcium signaling and secondary wall development.

We are deeply grateful to all of the authors, reviewers, and *ad hoc* editors for their contributions to the success of this Research Topic. We trust that the research community will benefit from this collection of knowledge at the frontiers of cell wall biosynthesis, its regulation, and the impacts of the plant cell wall on plant development and physiology.

## Author Contributions

XR and LB drafted the manuscript. All authors revised and approved the final version.

## Conflict of Interest

The authors declare that the research was conducted in the absence of any commercial or financial relationships that could be construed as a potential conflict of interest.
